# A Crucial Role of ACBD3 Required for Coxsackievirus Infection in Animal Model Developed by AAV-Mediated CRISPR Genome Editing Technique

**DOI:** 10.3390/v13020237

**Published:** 2021-02-03

**Authors:** Hye Jin Shin, Keun Bon Ku, Soojin Kim, Heon Seok Kim, Yeon-Soo Kim, Bum-Tae Kim, Seong-Jun Kim, Chonsaeng Kim

**Affiliations:** 1 Center for Convergent Research of Emerging Virus Infection, Korea Research Institute of Chemical Technology, Daejeon 34114, Korea; shinhy@krict.re.kr (H.J.S.); kbku@krict.re.kr (K.B.K.); btkim@krict.re.kr (B.-T.K.); 2 Graduate School of New Drug Discovery and Development, Chungnam National University, Daejeon 34134, Korea; support@icgm.kr (S.K.); kimys58@cnu.ac.kr (Y.-S.K.); 3 Division of Oncology, Department of Medicine, Stanford University School of Medicine, Stanford, CA 94305, USA; heonseokkim@gmail.com

**Keywords:** ACBD3, coxsackievirus, in vivo genome editing, adeno-associated virus, Cas9 TG mouse

## Abstract

Genetic screens using CRISPR/Cas9 have been exploited to discover host–virus interactions. These screens have identified viral dependencies on host proteins during their life cycle and potential antiviral strategies. The acyl-CoA binding domain containing 3 (ACBD3) was identified as an essential host factor for the Coxsackievirus B3 (CVB3) infection. Other groups have also investigated the role of ACBD3 as a host factor for diverse enteroviruses in cultured cells. However, it has not been tested if ACBD3 is required in the animal model of CVB3 infection. Owing to embryonic lethality, conventional knockout mice were not available for in vivo study. As an alternative approach, we used adeno-associated virus (AAV)-mediated CRISPR genome editing to generate mice that lacked ACBD3 within the pancreas, the major target organ for CVB3. Delivery of sgRNAs using self-complementary (sc) AAV8 efficiently induced a loss-of-function mutation in the pancreas of the Cas9 knock-in mice. Loss of ACBD3 in the pancreas resulted in a 100-fold reduction in the CVB3 titer within the pancreas and a noticeable reduction in viral protein expression. These results indicate a crucial function of ACBD3 in CVB3 infection in vivo. AAV-mediated CRISPR genome editing may be applicable to many in vivo studies on the virus–host interaction and identify a novel target for antiviral therapeutics.

## 1. Introduction

The type II clustered regularly interspaced short palindromic (CRISPR) system has been used for gene-specific modification and functional genetic screens [1,2,3,4,5]. Cas9 can be combined with single-guide RNAs (sgRNAs) to generate DNA double-strand breaks, which activate the DNA repair system for insertions or deletions (indels) of DNA sequences. This genetic perturbation introduced the loss-of-function mutations and revealed the functional role of specific genes. The CRISPR/Cas9-based genome editing system has been applied in a wide variety of experimental models, including cell lines, laboratory animals, plants, and even in human clinical trials [6]. In vivo genome editing has been performed to validate models of human diseases and to test the potential therapies using viral delivery systems of CRISPR/Cas9 [7,8]. Adeno-associated virus (AAV) vector systems have been used mainly due to their excellent safety profile, low immunogenicity, and stable transgene expression [9,10,11]. As many serotypes of AAV have different tissue specificity, tissue-targeted delivery of Cas9/sgRNA is also possible [12]. The AAV vector systems contain a single-stranded (ss) genome that is converted to a double-stranded (ds) form prior to transgenic expression. This conversion is a slow process responsible for delayed transduction and inefficiency [13,14]. The AAV vector systems containing self-complementary (sc) genomes (scAAV) were developed to circumvent the conversion into dsDNA, and these systems improved the kinetics of gene expression [15]. However, the AAV vector system has limited packaging capacity, and it is challenging to package the Cas9 along with sgRNA. To facilitate more versatile applications in in vivo mouse experiments, a Cas9 knock-in mouse was developed [16]. These Cas9-expressing mice showed no toxicities and tolerated the overexpression of the Cas9 protein [17]. This mouse line, along with AAV, expressing only sgRNAs, can be applied to achieve loss-of-function mutations in murine tissues efficiently without the limitation of a packaging capacity.

Genetic screens using CRISPR/Cas9 have been exploited to discover host–virus interactions [18]. These screens have identified viral dependencies on host proteins during their life cycle and potential antiviral strategies. The strong agreement of host factors from independent screens proved the validity of this technique. We performed the CRISPR screens to identify host factors required for enteroviruses causing diverse illnesses in humans [19,20]. Based on an arrayed CRISPR screen with an image-based assay, the acyl-CoA binding domain containing 3 (ACBD3) was identified as an essential host factor for the Coxsackievirus B3 (CVB3) infection. ACBD3-knockout cells were resistant to CVB3 and blocked viral replication altogether [20]. There have been many other reports investigating the role of ACBD3 in in vitro cell culture models and structural analyses [21,22,23,24,25,26]. However, it has not been tested whether ACBD3 is required in the animal model of CVB3 infection. A strong understanding of the host factors can be obtained when in vitro results are complemented by in vivo experiments. Knockout mice are preferred to explore the role of host factors in an animal model of a viral infection. Several groups have used the knockout mice compared with the wild-type mice to find the host proteins that are required for the pathogenesis and tropism of the virus [27,28,29,30]. It has been reported that ACBD3 is essential for embryonic development and ACBD3 knockout embryos die between 8.5 and 10.5 days of age [31]. Therefore, an alternative approach to obtain the ACBD3-deficient mice is required, different from the conventional method.

In this study, we aimed to obtain the ACBD3-deficient mice using AAV-mediated CRISPR genome editing. sgRNAs targeting mouse ACBD3 were delivered into transgenic mice expressing Cas9 by AAV to induce loss-of-function mutations on ACBD3. As the pancreas is one of the major target organs of CVB3 in a mouse infection [32] and AAV serotype 8 (AAV8) induces an efficient gene transfer in the adult mouse pancreas by the intraperitoneal delivery method [33], AAV8 was chosen to deliver sgRNAs into the pancreas. After confirmation of the knockout efficiency on ACBD3 in the pancreas, we suggest that this host factor is essential for CVB3 infection in an in vivo model.

## 2. Materials and Methods

### 2.1. Viruses and Cells

Coxsackievirus B3 (strain H3) was a generous gift of Dr. Eun-Seok Jeon (Samsung Medical Center, Seoul, Korea). CVB3-H3 was expanded and titered using HeLa cells [34]. A large-scale single-stranded AAV serotype 8 vector (ssAAV8) was provided by ViGene Biosciences (Rockville, MD, USA). The ssAAV vector was constructed based on selected sgRNA sequences. Viral production and titration were performed by ViGene Biosciences HeLa cells (ATCC, CCL2), and NIH/3T3 cells (ATCC, CRL-1658) were purchased from ATCC. These cells were maintained in Dulbecco’s Modified Eagle Medium (DMEM) (Hyclone, San Angelo, TX, USA) supplemented with 10% fetal bovine serum (Hyclone, San Angelo, TX, USA).

### 2.2. sgRNA Design

sgRNA sequences were designed using the Cas-Designer (http://www.rgenome.net/cas-designer/) based on the mouse reference genome mm10 [35]. sgRNAs with fewer potential off-target sites and higher microhomology scores were selected to optimize the mutation rate and specificity.

### 2.3. Mutation Analysis by Targeted Deep Sequencing

Genomic DNA was extracted using the DNeasy Blood and Tissue kit (Qiagen, Dusseldorf, Germany) according to the manufacturer’s instructions. For analysis of the mutations, sgRNA target sites were amplified with the KAPA HiFi HotStart PCR kit (KAPA Biosystems, Wilmington, MA, USA, KK2501) using 50 ng of the genomic DNA as the template as per a previously described method [36]. Sequencing output was analyzed using the Cas-analyzer (http://www.rgenome.net/cas-analyzer/) to determine the mutation rate [37]. Default parameters were used for analysis.

### 2.4. scAAV8 Vector Construction and Viral Production

The GFP sequence of the plasmid scAAV-GFP (CELL BIOLABS, San Diego, CA, USA, VPK430) was replaced by DsRed2 for constructing scAAV-DsRed2. The fragments of sg-Cont and sg-ACBD3 in the ssAAV plasmids from ViGene Biosciences were inserted into the plasmid scAAV-DsRed2. Three plasmid vectors were used to produce scAAV8: (1) an scAAV vector containing sg-Cont or sg-ACBD3 flanked by the inverted terminal repeat (ITRs) (delta D sequence in right ITR); (2) a packaging vector, AAV Rep2-Cap8 containing the AAV Rep2 and Cap8 genes (Addgene, 112864); and (3) a helper vector, pHelper (CELL BIOLABS, San Diego, CA, USA, VPK430) containing the adenovirus helper functions. The HEK293T cells in one CF10 (Sigma-Aldrich, St. Louis, MO, USA, Z720895) were co-transfected with pHelper, scAAV, and AAV Rep2-Cap8 plasmids using PEIpro (Polyplus-transfection, Illkirch-Graffenstaden, France, 115–100). At 72 h post-transfection, the transfected cells were collected and resuspended in the AAV Lysis Buffer (150 mM NaCl, 50 mM Tris-HCl pH 8.0). The suspensions were subjected to 3 freezing–thawing cycles to lyse the cells and incubated with benzonase (Sigma-Aldrich, St. Louis, MO, USA, E1014-5KU) at 37 °C for 1 h. The lysates were clarified by centrifugation. For in vivo use, scAAV8 was further purified using a discontinuous iodixanol gradient ultracentrifugation. The scAAV8 was desalted and concentrated with Amicon Ultra cell 100K filter units (Millipore, Burlington, MA, USA, UFC910024), aliquoted, and stored at −80 °C.

### 2.5. scAAV8 Titration

The titer of scAAV8 was determined by quantitative PCR with primers specific to the CMV promoter (CMV-F: 5′-TGACGTCAATGGGAGTTTGT-3′, CMV-R: 5′-GGCGGAGTTGTTACGACATT-3′) and SsoAdvanced Universal SYBR Green Supermix (Bio-Rad, Hercules, CA, USA, 1725270). A serial dilution of the plasmid scAAV-DsRed2 was used as a standard, and the copy number of the viral genome was measured using the CFX96 Touch Real-Time PCR Detection System and CFX Maestro Software (Bio-Rad, Hercules, CA, USA).

### 2.6. Cas9 Knock-in Mouse and In Vivo AAV Administration

Cas9 knock-in mice were obtained from Jackson Laboratories (Stock# 026179). All animal experiments were reviewed and approved by the Institutional Animal Care and Use Committee (IACUC) of the Korea Research Institute of Chemical Technology (2018-8A-11-01 and 2019-8B-04-01). Sex- and age-matched six-to-twelve-week-old Cas9 knock-in mice were divided into two groups. The first group was injected intraperitoneally with 5 × 10^11^ viral genome (vg) of AAV8 sg-ACBD3. The second group received the same dose of AAV8 sg-Cont. Two weeks after AAV8 infection, both groups were administered CVB3-H3 at 1 × 10^6^ TCID_50_ for 3 days by intraperitoneal injection.

### 2.7. Determination of the CVB3 Titer and Immunoblotting Using the Mouse Pancreas

Tissue samples from the pancreas were weighed and subsequently homogenized in the cold phosphate-buffered saline (PBS) solution with a FastPrep-24 homogenizer (MP Biomedicals, Irvine, CA, USA) for 5 cycles (20 s on/20 s off). Homogenized samples were divided for further experiments. Three additional freeze–thaw cycles were performed at −80 °C, and cell debris was removed by centrifugation at 13,000 rpm for 1 min to measure the viral titer in the pancreas. The HeLa cells were seeded in 96-well plates and infected with 10-fold serial dilutions of the viral supernatant. After 48 h of infection, a modified 3-(4,5-dimethylthiazol-2-yl)-2,5-diphenyltetrazolium bromide (MTT; Sigma-Aldrich, St. Louis, MO, USA) assay was performed as per a previously described method [38]. The TCID_50_ value was calculated using the Reed and Muench method [39].

### 2.8. Immunohistochemistry Examinations

The isolated pancreas was fixed using 10% neutral buffered formalin (BBC Biochemical, Mckinney, TX, USA, 0152) and then processed for paraffin embedding (Leica, Wetzlar, Germany, ASP300S). Five-micron-thick slices cut from the paraffin blocks were placed on glass slides. The sections were deparaffinized in xylene and rehydrated through graded alcohol. The deparaffinized section was boiled in the antigen retrieval buffer (Abcam, Cambridge, UK, 94681) for 5 min and incubated with a hydrogen peroxide blocking buffer (Abcam, Cambridge, UK, 127055) for 20 min at room temperature. The slides were stained with the ACBD3-specific rabbit antibody (Sigma-Aldrich, St. Louis, MO, USA, HPA015594) that cross-reacts with mouse ACBD3, and rabbit polyclonal anti-3C antibody [40] in dilution buffer (1% bovine serum albumin in Tris-buffered saline containing 0.1% Tween-20) overnight at 4 °C. The sections were subsequently incubated with an HRP-conjugated secondary antibody at room temperature for 1 h and developed with 3,3′-diaminobenzidine (Abcam, Cambridge, UK, 94665). All paraffin-embedded sections were counterstained with hematoxylin (BBC Biochemical, Mckinney, TX, USA, MA0101010) and mounted in a Shandon Synthetic Mountant (Thermo Fisher Scientific, Waltham, MA, USA, 6769007). Images were obtained with the Nuance Multispectral Imaging System FX fixed to the Olympus BX51 using Nuance 3.02 software.

## 3. Results

### 3.1. In Vitro Screening of sgRNAs Specifically Targeting the Murine ACBD3 Gene

**Figure 1:**
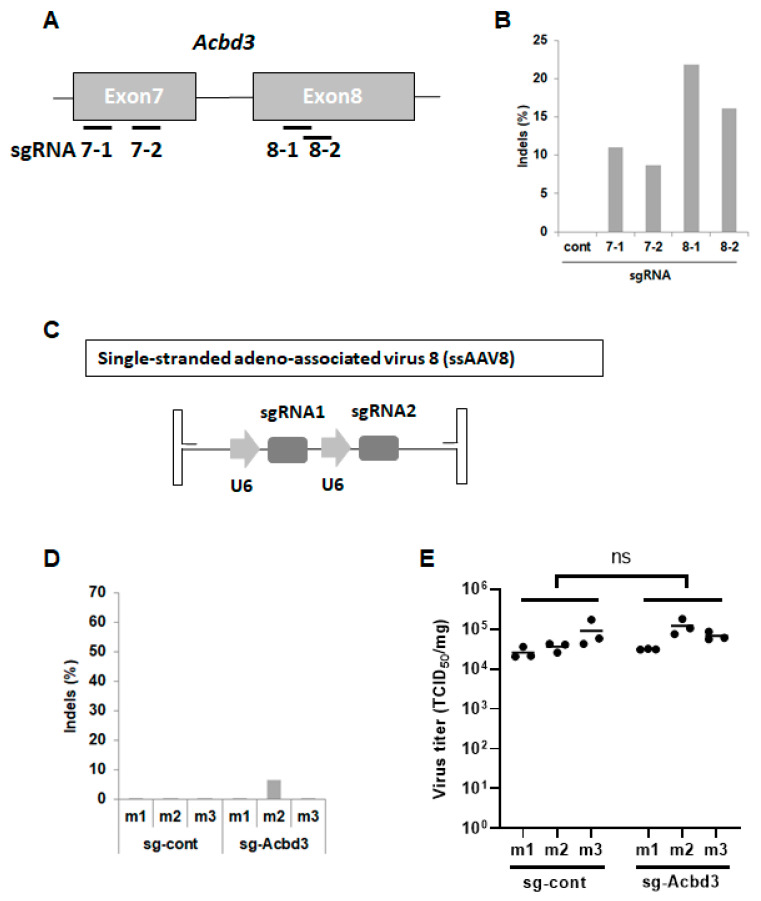
In vitro screening of sgRNAs targeting the murine ACBD3 gene and in vivo delivery into the Cas9 knock-in mice using single-stranded adeno-associated virus serotype 8 vector (ssAAV8). (**A**) sgRNA design for targeting the mouse ACBD3 locus. (**B**) ACBD3 indel analysis of mouse NIH/3T3 cells transfected with plasmids expressing sgRNAs along with the plasmid expressing Cas9. The results are shown as the percentage of sequencing reads containing indels at the target site. (**C**) A schematic diagram showing ssAAV8 vector construction expressing sgRNA1 and sgRNA2 under the U6 promoter. (**D**) ACBD3 indel analysis of the pancreas from the Cas9 knock-in mice injected with ssAAV8 expressing sg-Cont or sg-ACBD3. (**E**) Cas9 knock-in mice transduced with ssAAV8 expressing sg-Cont or sg-ACBD3 were infected with CVB3 for 3 days. CVB3 titers in the pancreas of the mice were determined as TCID_50_ per milligram of the tissue (*n* = 3). Statistical significance was calculated by an unpaired *t*-test using GraphPad Prism software. Ns indicates *p* > 0.05.

Recently, we have shown that sgRNAs targeting exon 7 and exon 8 of the human ACBD3 introduced the knockout mutation very efficiently [20]. To induce the mutations in the mouse ACBD3 gene, new sgRNAs targeting this gene are required. We designed four sgRNAs that targeted different sites of exon 7 (sgRNA 7-1/7-2) or exon 8 (sgRNA 8-1/8-2) in ACBD3 to select the highly efficient sgRNAs (Figure 1A). These four sgRNA expression plasmids were transfected into the NIH/3T3 mouse cells along with the Cas9 expression plasmid. Each sgRNA showed different indel rates depending on their position on the exon (Figure 1B). Mutations were introduced more efficiently using sgRNAs targeting exon 8 (sgRNA 8-1 and sgRNA 8-2). These two sgRNAs were chosen for further in vivo editing experiment.

### 3.2. Pancreatic ACBD3 Gene Editing in a Cas9 Transgenic Mouse Using Single-Stranded AAV8

To induce the ACBD3-knockout mutation in the pancreas, we first constructed two single-stranded AAV vectors (ssAAV), which expressed two sgRNAs targeting ACBD3 (sg-ACBD3) under the control of the human U6 promoter and two control sgRNAs (sg-Cont) as a control virus (Figure 1C). These vectors were packaged with the AAV serotype 8 vector (ssAAV8), which was previously shown to effectively infect the pancreas [33]. Two viruses were administered into the Cas9 transgenic mouse via intraperitoneal injection. Two weeks post-injection, CVB3 was infected intraperitoneally. The indel formations and virus titers in the pancreas were measured on day 3 after CVB3 infection. Deep sequencing of the pancreatic tissue showed a low editing efficiency, with less than 7% (Figure 1D). CVB3 titers in the pancreas were similar between sg-ACBD3 and sg-Cont injected mice (Figure 1E). These results suggest that the delivery of sgRNAs using ssAAV8 does not efficiently induce the mutations to investigate the effect of ACBD3 on the pancreatic infection of CVB3.

### 3.3. Loss of ACBD3 Using Self-Complementary AAV8 Attenuates Pancreatic CVB3 Infection

**Figure 2:**
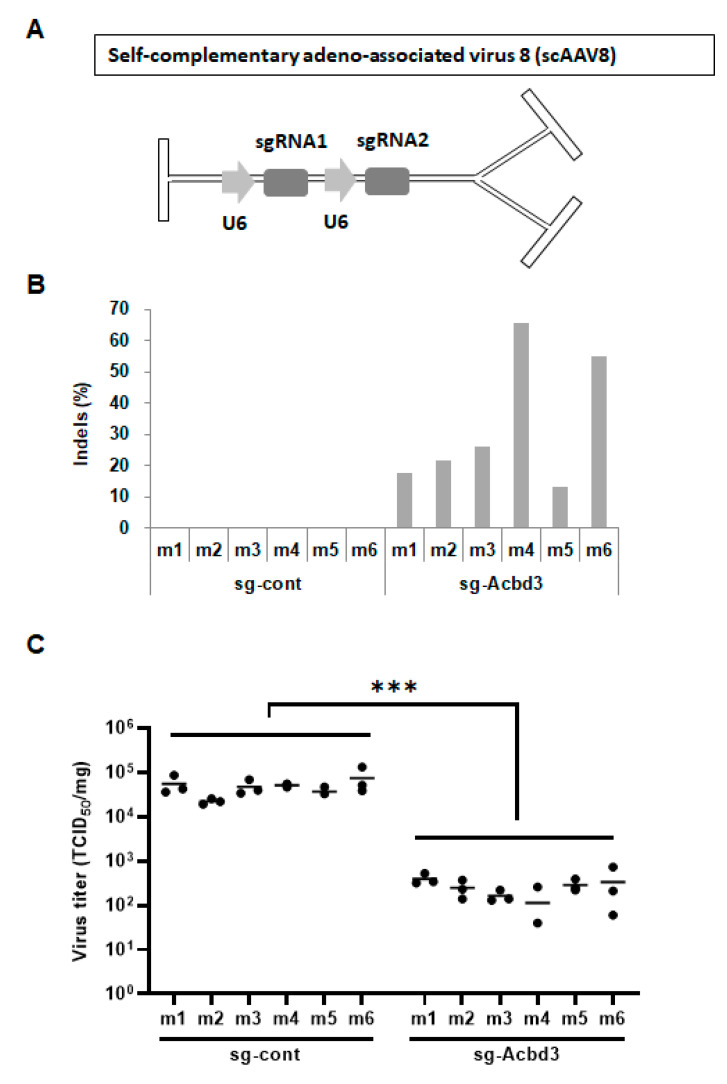
Reduced CVB3 infection in the pancreas of the ACBD3-deficient mice using scAAV8-expressing sg-ACBD3. (**A**) Schematic diagram of the scAAV8 vector. Vertical bars represent AAV inverted terminal repeats (ITRs). (**B**) ACBD3 indel analysis of the pancreas from the Cas9 knock-in mice injected with the scAAV8-expressing sg-Cont or sg-ACBD3. (**C**) Cas9 knock-in mice transduced with the scAAV8-expressing sg-Cont or sg-ACBD3 were infected with CVB3 for 3 days. CVB3 titers in the pancreas of mice were determined as a TCID_50_ per milligram of the tissue (*n* = 3). Statistical significance was calculated by an unpaired t-test using GraphPad Prism software. *** indicates *p* < 0.0001.

Previous reports have demonstrated more efficient gene delivery by self-complementary AAV (scAAV) than that by ssAAV in vitro and in vivo [13,14,15]. To improve the efficiency of pancreatic ACBD3 editing, we constructed the scAAV vectors expressing the same sgRNAs in the ssAAV vectors (Figure 2A). Two vectors expressing the control sgRNA (sg-Cont) and the ACBD3-targeting sgRNA (sg-ACBD3) were packaged using the AAV serotype 8 vector. Injection of scAAV8 and CVB3 into the Cas9 knock-in mice was performed using a method similar to the previous experiment using ssAAV8. Twelve mice were divided into two groups. The first group (*n* = 6) were injected with the scAAV8-expressing sgRNAs targeting ACBD3, and the second group (*n* = 6) were injected with control scAAV8. Both groups were infected with CVB3 for three days. The pancreas was dissected to measure the efficiency of gene editing by deep sequencing. In contrast to ssAAV8, the scAAV8-expressing sg-ACBD3 showed a higher genome editing efficiency from 13% indel ratio reaching up to 65% (Figure 2B). The control scAAV8 showed a marginal indel ratio (Figure 2B). We measured the productive CVB3 infection in these two groups. As shown in Figure 2C, pancreas titers in ACBD3-edited mice (sg-ACBD3) were more than 100-fold lower than that of the control-edited mice (sg-Cont). Strong inhibition of CVB3 infection was detected in vivo with the increased efficiency of ACBD3 editing using scAAV8.

**Figure 3:**
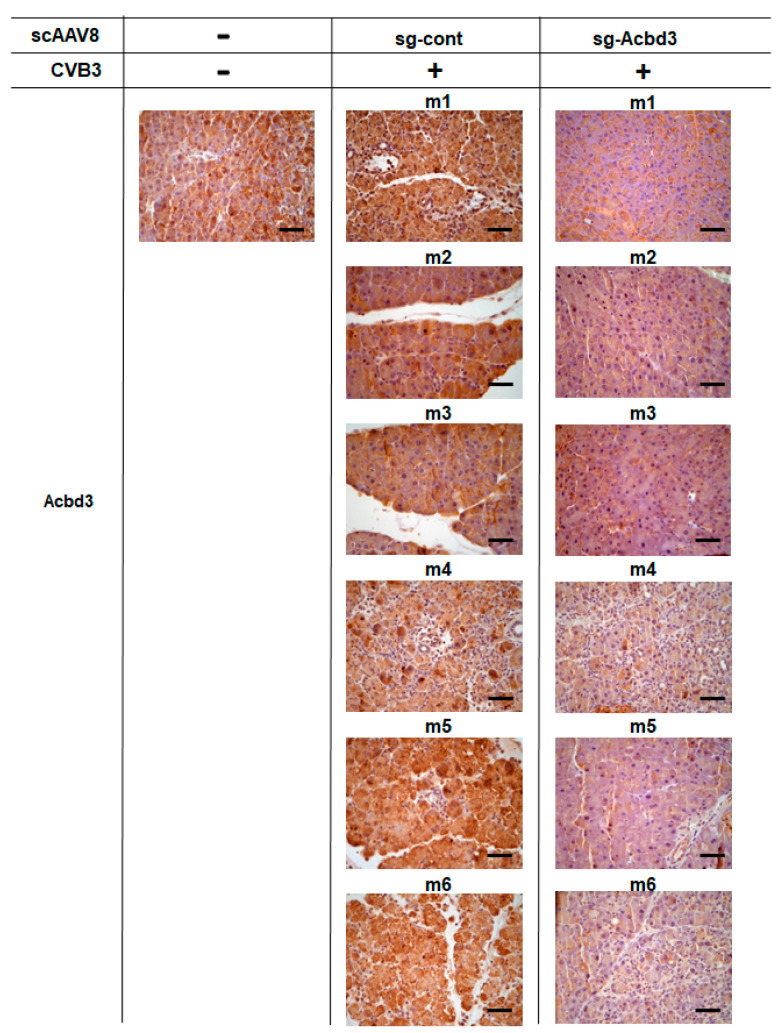
Immunohistochemical staining of ACBD3 in the pancreas of the Cas9 knock-in mice. Immunohistochemical staining of the pancreatic tissue was performed using an anti-ACBD3 antibody. Representative images of Cas9 knock-in mice without the scAAV8 injection and CVB3 infection, Cas9 knock-in mice injected with the scAAV8-expressing sg-Cont and infected with CVB3, and Cas9 knock-in mice injected with the scAAV8-expressing sg-ACBD3 and infected with CVB3. Bars, 100 µm.

**Figure 4:**
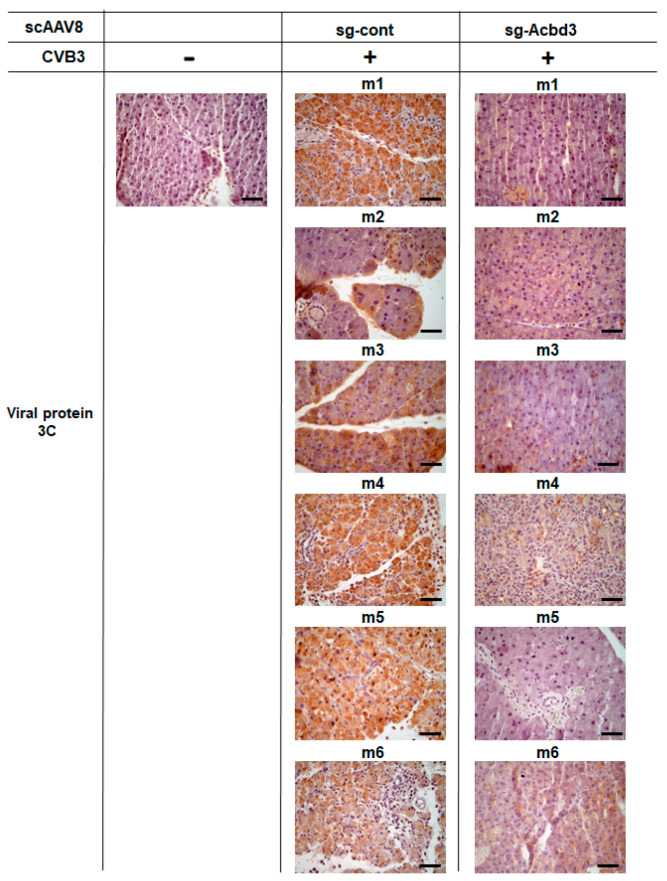
Immunohistochemical staining of the viral protein 3C in the pancreas of the Cas9 knock-in mice. Immunohistochemical staining of the pancreatic tissue was performed using the antiviral protein 3C antibody. Representative images of Cas9 knock-in mice without the scAAV8 injection and CVB3 infection, Cas9 knock-in mice injected with the scAAV8-expressing sg-Cont and infected with CVB3, and Cas9 knock-in mice injected with the scAAV8-expressing sg-ACBD3 and infected with CVB3. Bars, 100 µm.

To further confirm the depletion of ACBD3, we performed immunohistochemical staining of the pancreatic tissue. The Cas9 knock-in mice without scAAV8 and CVB3 injection showed intense brown staining of ACBD3 (Figure 3). Mice in the sg-Cont group showed similar staining of ACBD3 as that in the control mice (Figure 3). The decreased expression of ACBD3 was detected in the sg-ACBD3 group (Figure 3). Similar immunohistochemical staining of the pancreatic tissue was performed to confirm the expression of viral protein 3C. No staining of the 3C protein was detected in the control Cas9 knock-in mice (Figure 4). Strong brown staining of the 3C protein was observed in mice from the sg-Cont group (Figure 4). The staining of the 3C protein was decreased in mice from the sg-ACBD3 group (Figure 4). These staining results of the 3C protein matched well with those measured for the viral titer, as shown in Figure 2C. Taken together, depletion of ACBD3 mediated by sgRNA-expressing scAAV8 inhibited the pancreatic infection of CVB3 in the Cas9 transgenic mice. These results suggest that ACBD3 is an essential host factor for in vivo CVB3 infection.

## 4. Discussion

In this study, we report that ACBD3 is an essential host factor for a CVB3-mediated pancreatic infection in vivo. Efficient induction of the loss-of-function mutation was successful using scAAV8 for the delivery of ACBD3-targeting sgRNAs into the Cas9 knock-in mice. Our results also showed that scAAV was more efficient in gene transfer than the efficiency exhibited by ssAAV as described in previous reports [13,14]. Loss of ACBD3 in the pancreas resulted in a 100-fold reduction in CVB3 titer, and strong inhibition of the viral protein expression was confirmed by immunoblot and immunohistochemical staining. To the best of our knowledge, this is the first report to determine ACBD3 as an essential host factor in a mouse model of CVB3 infection. Considering the dependency of diverse enteroviruses, including CVB3, on ACBD3 [25], our results suggest that ACBD3 is required for an in vivo infection by pan-enterovirus.

ACBD3 may be a novel target to develop broad-spectrum anti-enterovirus therapeutics. In vivo viral dependency of ACBD3 further strengthens this possibility. It will be very challenging to develop a human gene therapy using the CRISPR/Cas9 technology, which introduces knockout mutations in ACBD3, to prevent the viral infection owing to many hurdles that need to be overcome. The 3A protein of enteroviruses interacts with ACBD3 to recruit other cellular proteins for viral replication [41,42]. It is more feasible to develop the compounds targeting ACBD3 or the 3A-ACBD3 interactions. These compounds may temporarily inhibit viral propagation without leaving permanent genetic mutations.

**Figure 5:**
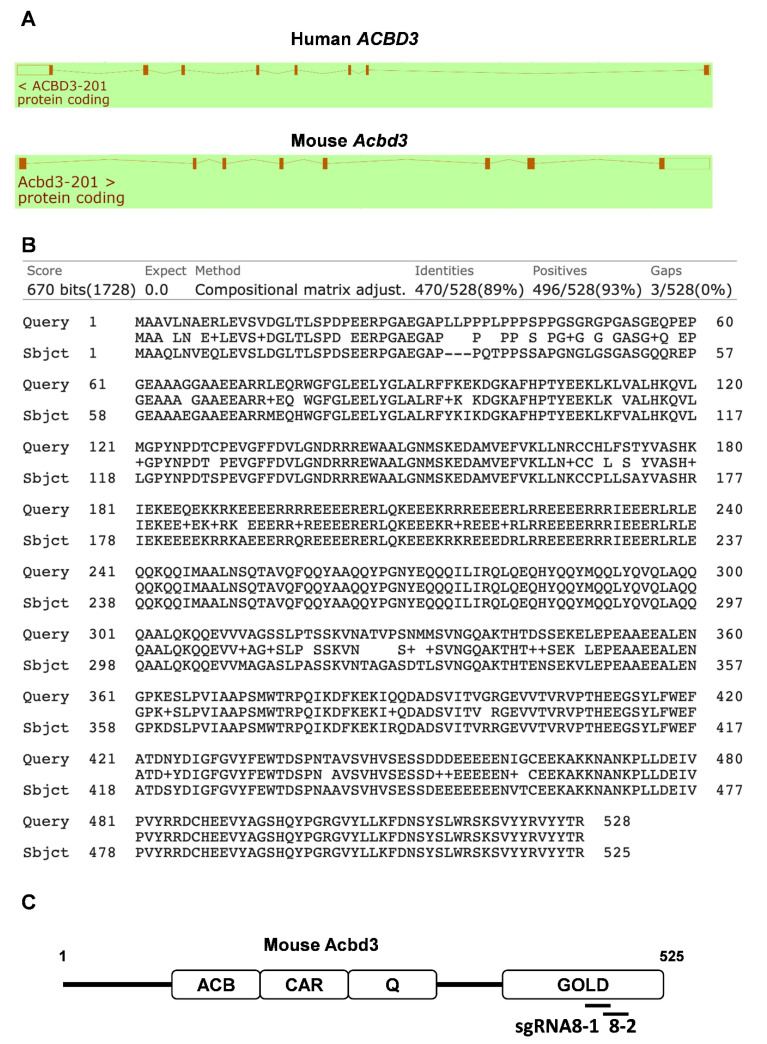
Comparison of the human *ACBD3* and mouse *ACBD3* gene. (**A**) Schematic diagram of the intron–exon structure for the human *ACBD3* and mouse *ACBD3* genes generated by Ensembl browser (www.ensembl.org). (**B**) Blast homology search between human ACBD3 (Query) and mouse ACBD3 (Sbjct) proteins (blast.ncbi.nlm.nih.gov). (**C**) Schematic diagram of the functional domains of ACBD3. The target sites of sgRNAs are depicted at the bottom.

ACBD3 is a Golgi scaffolding protein and is known to bind palmitoyl-CoA and PI4KB proteins [43]. Both human ACBD3 and mouse ACBD3 genes were shown to consist of 8 exons (Figure 5A). In amino acid comparison between human ACBD3 and mouse ACBD3 proteins, these two proteins have a high similarity of 89% identity (Figure 5B). Based on the functional domains of human ACBD3 [25], the schematic diagram showed four functional domains of ACBD3 (Figure 5C). The Q and GOLD domains were required for enterovirus replication, but the ACB and CAR domains were dispensable [25]. As our sgRNAs targeting exon 8 introduced mutations in the GOLD domain (Figure 5C) required for CVB3 replication, the expression of ACBD3 with the truncated GOLD domain could mediate the reduction in pancreatic viral titers.

Currently, in vivo tests to determine the viral host factors rely on the knockout mice [28,29,30]. However, knockout of the proteins essential for embryogenesis, such as ACBD3 in mice, has proved to be lethal. Therefore, it is impossible to study these proteins using the conventional gene knockout methods. Conditional-knockout mice generated by the Cre-loxP technology have been used to delete the gene at a specific time and tissue to bypass the problem of embryonic lethality. These mice have also been used to study virus–host interactions [27,44]. However, it is cumbersome and takes a long time to generate these knockout mice. The CRISPR/Cas system has considerably increased the feasibility and ease of editing the mouse genome [45]. In vivo editing of somatic tissues by the CRISPR/Cas system provides alternative methods to generate a mouse model with a gene deletion, saving significant time and effort. Here, we proved that this alternative approach was an efficient way to determine the importance of viral host factors during in vivo infection of the virus using CVB3 and ACBD3 as a model system. Our findings propose that the application of AAV-mediated CRISPR genome editing will be an effective strategy to examine the role of viral host factors in the mouse system.

## Data Availability

The data presented in this study are available within this article.
